# Comparative analysis on visual and olfactory signals of *Papilio xuthus* (Lepidoptera: Papilionidae) during foraging and courtship

**DOI:** 10.1371/journal.pone.0263709

**Published:** 2022-06-29

**Authors:** Jie Liu, Mingtao Li, Shunan Chen, Jun Yao, Lei Shi, Xiaoming Chen

**Affiliations:** Key Laboratory of Breeding and Utilization of Resource Insects of State Forestry Administration, Institute of Highland Forest Science, Chinese Academy of Forestry, Kunming, P.R. China; University of Mississippi, UNITED STATES

## Abstract

This study examined the roles of visual and olfactory responses during foraging and courtship in butterfly *Papilio xuthus*. *P*. *xuthus* showed obvious orientation to color in the range of 350–500 nm. Visits of *P*. *xuthus* females and males to blue, purple, and red artificial cloth flowers were ♀ 54.90% and ♂ 39.22%, ♀ 19.61% and ♂ 35.29%, and ♀ 9.80% and ♂ 19.61%, respectively. Application of 10% honey on these artificial flowers resulted in an increase of 3.41 and 3.26 fold in flower visits by the butterfly compared to controls. When 10% honey water was sprayed on flower branches without colorful flowers, branch visiting was very low, only seven times for females and two times for males, indicating that colors might be more critical than odor for foraging even though visual and olfactory perceptions both play important roles during foraging. During courtship, four types of chasing were observed in a natural population of *P*. *Xuthus*; the four types are males chasing females (49%), males chasing males (25%), females chasing males (13%), and females chasing females (10%). However, when odorless artificial models of butterflies were used, no significant differences were observed among these types of chasing, indicating that olfactory perception was crucial for the butterfly during courtship. Profiling volatile organic chemicals (VOCs) and individual bioassays revealed that VOCs contents of butterflies were not related to recognizing sex partners; by contrast, some level of α-farnesene, increased the frequency of male chasing female. This could be due to that α-farnesene is easy to be detected by butterflies because of its volatility and higher content in female.

## Introduction

Visual and chemical cues are critical during butterfly foraging and courtship, but their roles vary in individual butterfly species. The eyes of most butterflies contain visual pigments that can sense a range of the light spectrum including ultraviolet (300–400 nm), blue (400–500 nm), long -wave (500–600 nm) part of the visible light spectrum [1]. During foraging, different taxa of butterflies use different visual cues to locate food sources. Most butterflies prefer blue [2], and some butterflies of Papilio and Pieris species prefer purple. *Tirumala limniace* and some Pieris and Vanessa prefer yellow, whereas *Danaus chrysippus*, *Cethosia biblis*, and *Cethosia cyane* are more sensitive to red [2–8]. During courtship, butterflies distinguish females from males of the same species primarily based on visual signals, including wing characteristics (color, spots, and size), wing surface reflection spectrum, and polarized light. Dichroic butterflies identify the opposite sex by the color of wings under natural light [9, 10]. *Pieris rapae* males identify females using visual cues of their wings [11]. *Heliconius cydno* males recognize females by their polarized aurora [12].

Chemical cues are also important in localizing food sources [13–16]. Butterflies can also distinguish the opposite sex from the same species and individuals from heterogenetic species based on chemical cues [17–20]. Some butterflies, such as *Kallima inachus* and *Danaus genutia*, have less developed visual systems. Therefore, chemical signals are their only cues during foraging and courtship. Volatile organic compounds (VOCs) from individual butterflies are critical for them to distinguish the opposite sex from the same species and individuals from different species [21–23]. For example, VOCs from the females and males of *Papilio machaon* are significantly different and serve as important cues in identifying the opposite sex from the same species [24].

Four types of relationships between visual and chemical communication have been identified during butterfly foraging and courtship: (1) vision was given priority over olfaction; (2) olfaction was given priority over vision; (3) olfaction and vision were equally important; and (4) the only olfaction was used. However, what determines the contribution of visual and chemical perception in foraging and courtship is not yet clear. *Papilio xuthus* (Lepidoptera, Papilionidae) is common species in China, Japan, Korea, and Myanmar, five generations each year in China; its main host plants are *Citrus reticulate*, *Zanthoxylum*, and *Zanthoxylum ailanthoides* [25]. In China, *P*. *xuthus* is used in butterfly gardens for tourism and the popularization of science by artificial breeding.

In this study, we conducted a series of assays to determine the importance of visual and chemical cues for the butterfly *P*. *xuthus* during foraging and courtship. We used butterfly models to separate the impact of visual and chemical cues on butterfly foraging and mating behavior. We also analyzed wing color patterns between the females and males, profiles of VOCs from males and females, and tested selected individual VOCs on butterfly behavior. Our results represent an initial analysis of the contribution of visual and chemical cues to foraging and mating behavior in this butterfly species.

## Materials and methods

All applicable international, national, and/or institutional guidelines for the care and use of animals were followed. All procedures performed in studies involving animals were in accordance with the ethical standards of the institution or practice at which the study was conducted.

### Test site

Observation was carried out in a net house (8 × 8 × 5 m) in Kunming (102° 45 ′ E, 25° 03 ′ N), Yunnan Province, southwestern China. The altitude was 1930 m. The annual average temperature was 15–16°C. The annual average rainfall was 900–1000 mm. The temperature was 23–30°C at the test site during the experiments.

### Test butterfly

*Papilio xuthus* was obtained from artificial breeding populations and used for the observation. Larvae were raised on young leaves of *Citrus reticulata* under controlled conditions at 27°C with a 13:11 (L: D) cycle, and 50–70% relative humidity. Adults for two days after emergence were used in the foraging assays, and only virgin adults for three days after emergence were used in the courtship behavioral assays.

### Artificial flowers

**Fig 1:**
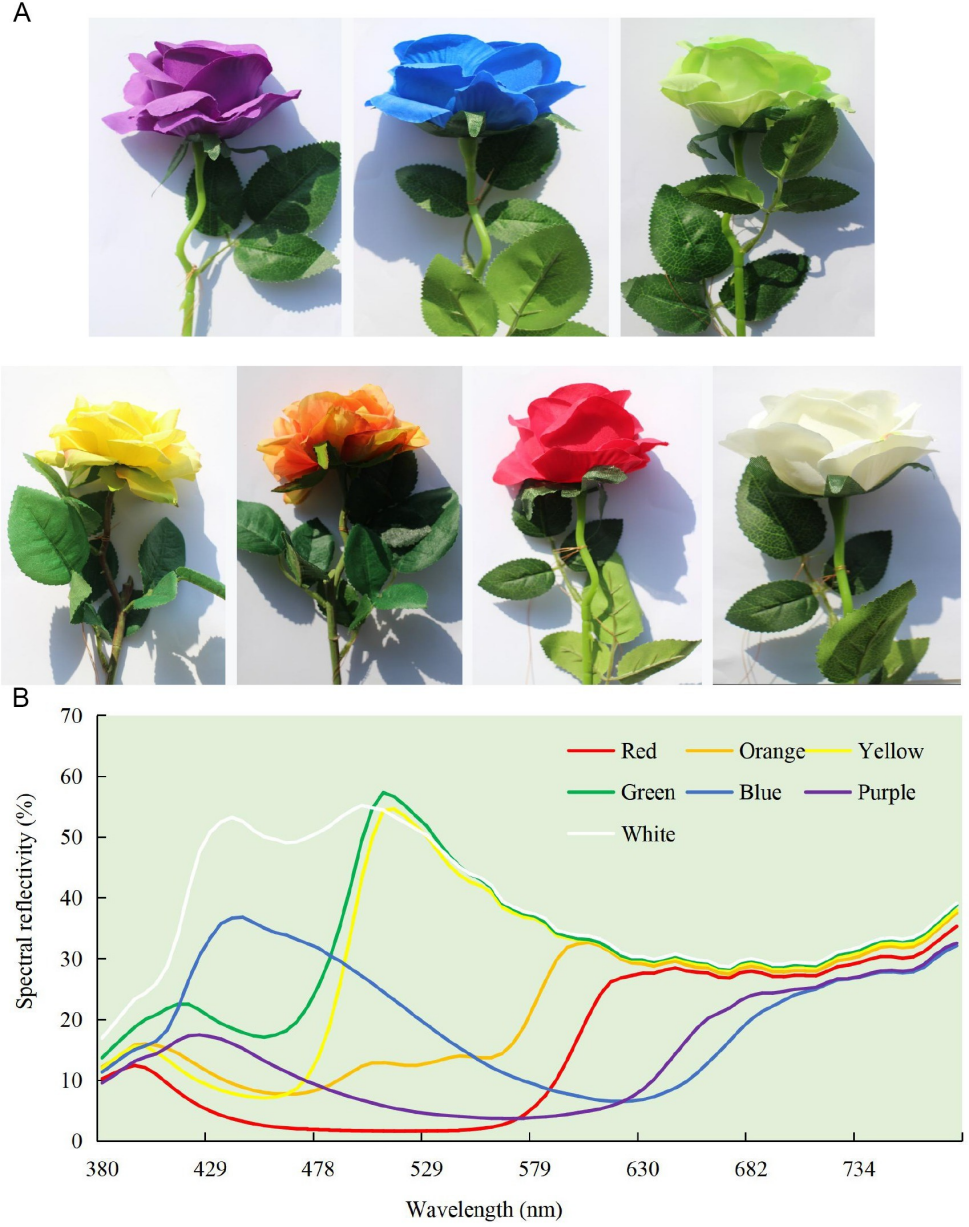
Artificial flowers and their spectral reflectance. A. Artificial flowers with seven colors; B. Spectral reflectance of the seven colors.

Artificial cotton flowers of seven colors were used for the test. The seven colors were red, orange, yellow, blue, purple, green, and white corolla. The size (8.56 ±0.18 cm in diameter and 2.73 ±0.04 cm in depth; Fig 1A) and shape of the artificial flowers were the same. A spectrometer (USB2000+, Ocean Optics, Inc., USA) was used to measure the reflection spectrum of flower colors. The spectrometer was calibrated against a MgO-coated surface as a reference, and the spectral reflection of the fake flowers was in the wavelength range of 380–780 nm (Fig 1B).

### Honey water

Honey made from acacia was diluted with deionized water to a 10% concentration [2].

### Butterfly models

**Fig 2:**
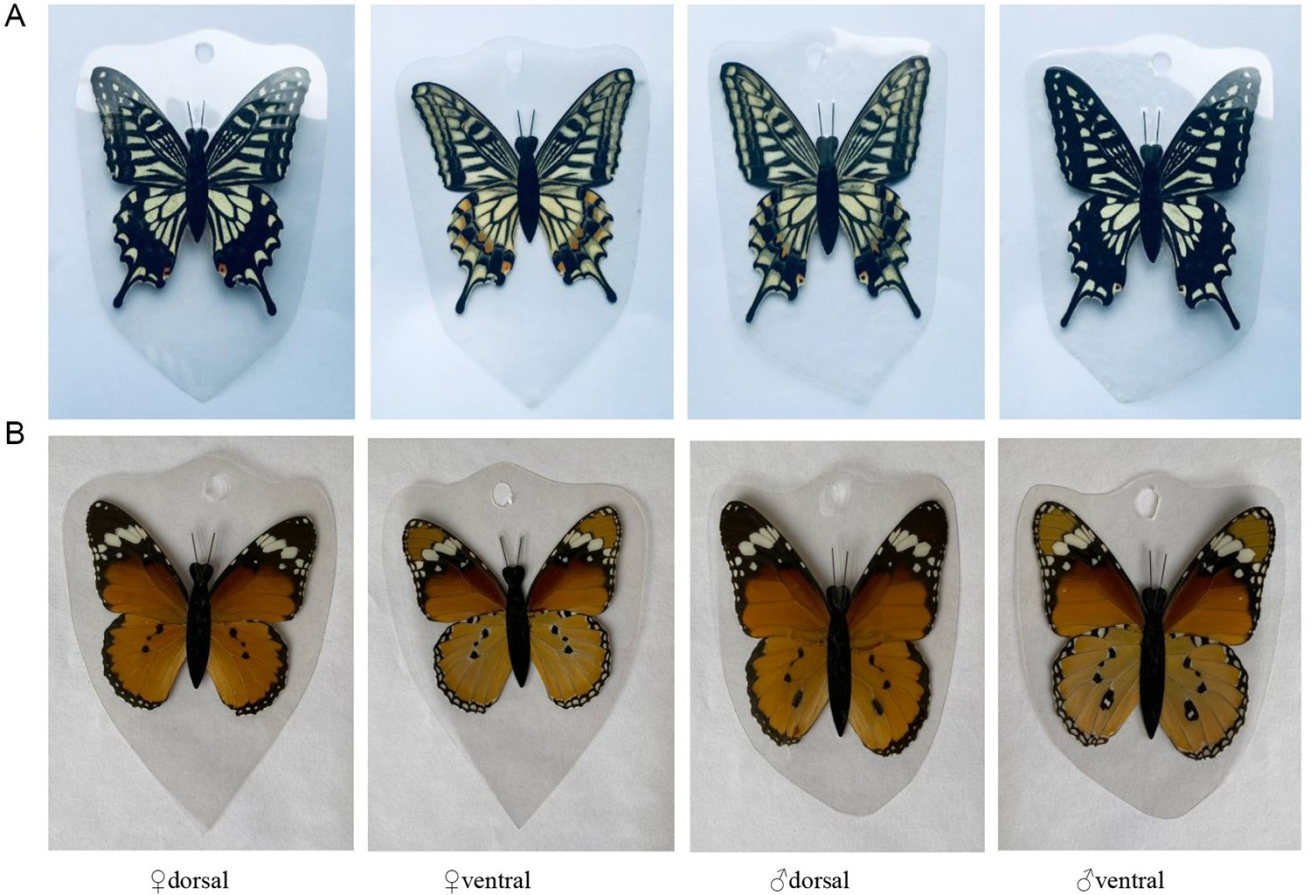
Artificial butterfly model. A. Artificial *P*. *xuthus* butterfly model. B. An artificial *D*. *chrysippus* butterfly model.

The real butterfly wings were attached to an insect body made of black plastic strips (2.0×0.6 cm) according to the dorsal and ventral surface, and then glue the false black antennae, finally covered by transparent plastic film. The butterfly models were the same in size as natural butterflies (Fig 2).

### Reflection measurements of natural butterflies

The reflection characteristics of the dorsal and ventral surfaces of the forewing and hindwing of natural butterflies were measured using a USB2000+ spectrometer (170 ms integration time, 30 averaged scans), PX-2 pulsed xenon light source, and magnesium oxide (MgO) reflectance standard. Reflectance was captured from a circular region 5 mm in diameter, with the wing rotated on a dual-axis stage to locate the orientation providing maximum brightness according to well-established protocols [10]. UV color determinations were performed using an ultraviolet spectrometer (WD-9403C, Beijing Liuyi Co., Beijing, China, with a peak wavelength of 365nm).

#### Experimental design

*Measurement of color attraction*. Seven artificial flowers in a nosegay were placed 0.5 m above the ground, and the distance between adjacent bouquets was 80 cm. Female and male butterflies of *P*. *xuthus* (10 pairs each) were released into the net house, and their behavior for visiting the fake flowers of seven colors was observed. The position of these flowers was moved clockwise once every 30 min.

According to the activity characterization of this butterfly, all behaviors were observed from 09:00 to 18:00. All experiments were repeated thrice, and the observation were made manually.

*Measurement of odor attraction*. Corollas from artificial flowers were removed, leaving only their branches and leaves. Honey water (10%, 15 ml) was then sprayed on these branches and leaves, and butterfly response to the chemical was examined and recorded.

*Measurement of the combined impact of both visual and chemical cues*. On the artificial flower bouquets, 15 ml of 10% honey water was sprayed, and flower visits of male and female butterflies were recorded. Honey water was sprayed every hour to avoid its evaporation.

Visitation frequency was recorded as the number of butterflies that landed on an artificial flower, extended the proboscis, and fed. If a butterfly fed on a flower and then returned and fed a second time on the original flower without flying at least 1 m away, this second visit was not counted.

#### Observation of courtship behavior

*Natural population courtship*. In the net house, 10 pairs of *P*. *xuthus* (♀:♂ = 1: 1) were released into the net house to observe their courtship behavior. Additionally, 10 pairs of another butterfly species, *Danaus chrysippus*, were also released to the same net house to determine any inter-species courtship between *P*. *xuthus* and *D*. *chrysippus*.

Chasing number was recorded as the number of butterflies that a butterfly flew and chased another butterfly during adult courtship.

*Butterfly chasing models*. To separate impact of visual and chemical cues on butterfly behavior during courtship, models of *P*. *xuthus* and *D*. *chrysippus* were used to test the discrimination of *P*. *xuthus* based only on butterfly models colors. Four pairs of butterfly models (♀:♂ = 1: 1) for each species were hung randomly at the height of 1.5~2.0 m above the ground with 80 cm apart from each other. Then 10 pairs of virgin *P*. *xuthus* butterflies (♀:♂ = 1: 1) were released into the net house, and their chasing behavior toward the models was recorded. The models were rotated clockwise every 30 min. The experiment was divided into two ways: Mix released models: 10 pairs of virgin *P*. *xuthus* butterflies (♀:♂ = 1: 1) were placed in these wet houses at once; Respectively released models: male and female butterflies were placed separately.

The number of butterfly chasing models was recorded as the number of butterflies that a butterfly approaches the moving model, flying around or staying on the model.

*Measurement of Volatile Organic Chemicals (VOCs)*. To measure the impact of volatiles, the method solid-phase microextraction (SPME) was used to extract VOCs from female and male butterflies separately. The samples were then analyzed using GC-MS (Thermo Scientific TRACE GC ULTRA and Thermo Scientific ITQ 900 MS; Borg-Karlson & Mozuraitis, 1996). When mating started, *P*. *xuthus* individuals were put into an 80 mL wide-mouth bottle two days after eclosion. A PDMS/DVB extraction needle with 65 μm diameter (Supelco, Bellefonte, PA, USA) was then inserted into the bottle for 1 h. A SPME extraction needle was inserted at the GC inlet (250°C, 10 min) before GC-MS analysis. A TR-5MS column, with an inner diameter 0.25 mm, thickness 0.25 μm, and length 30 m, was used to analyze VOCs. The program for GC-MS analysis was 2 min at 40°C, 2 min at 120°C (achieved by heating at 4°C /min), and 5 min at 230°C (by heating 5°C /min). The inlet temperature was 250°C, and the pressure of the carrier gas He was 69 kPa.

Identification of VOCs was carried out by searching the National Library of Standards and Technology (NIST, http://webbook.nist.gov/chemistry/) library combined with a manual analysis of mass spectra. Retention indices were calculated and compared with corresponding values in the literature. The retention index (linear retention index, LRI or RI) was calculated as follows [10]:

$$LRI=100\times n+\frac{100\times (t_{x}-t_{n})}{t_{n+1}-t_{n}}$$

LRI-retention index;

*t*_*x*_*—*retention time of the component to be tested (min);

*t*_*n*_*—*retention time of n-alkanes with n carbon atoms(min);

*t*_*n+*1_—Retention time of n-alkanes with n+1 carbon atoms(min).

The area normalization method was used to determine the relative content of each volatile compound.

### Analysis of selected VOCs on butterfly behavior

In twenty-eight of VOCs, we selected the most content of abundant chemicals, 2-ethylhexanol (alcohol, 35%–37%), 2-ethylhexyl acetate (ester, 31%–35%), and α-farnesene (Terpenoid, 2–5%) (Fig 5D), were used to analyze of their impact on the courtship behavior of butterfly males and females. A 1% solution of each chemical was prepared, and 0.5 mL of each solution was injected into a 0.6 mL finger tube, attached to a butterfly model. Butterfly models were hung 1.8 m above the ground in the net house, and each model was placed one meter apart from the other. Ten pairs of virgin butterflies three days after eclosion were released into the net house. Their chase behaviors toward the butterfly models were observed and recorded. The butterfly models without chemicals were used as controls. The models of *D*. *chrysippus* were also used for observation of possible inter-species attraction.

### Data analysis

The SPSS 18.0 software was used for data analysis. One-way ANOVA was used to identify significant differences between males and females during foraging and courtship events. Levene test method was used to make variance homogeneity [26], and the LSD method was used to make multiple comparisons when the variances were homogeneous. Nonparametric rank-sum test is used when variance is not homogeneous.

## Results

### Response to visual and chemical cues during foraging

**Fig 3:**
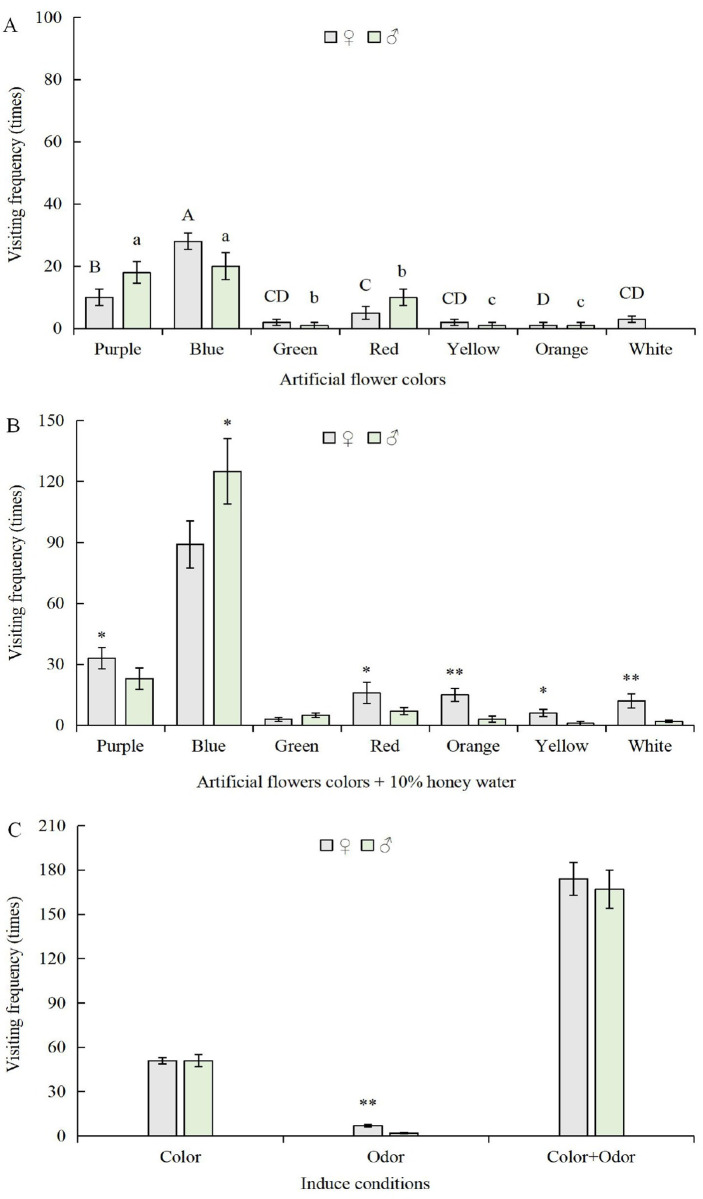
Response of the butterfly *P*. *xuthus* to artificial flowers of different colors and the combinations of different colors and odors. A. Response of *P*. *xuthus* to flowers with different colors. B. Response of *P*. *xuthus* to flowers sprayed with 10% honey water. C. Synergistic effects of colors and odor on butterfly behavior. Note: Capital letters indicate significant differences among the female visits to different flower colors (P <0.05) (N = 21; LSD method); lowercase letters indicate significant differences of male visits to different colors (P<0.05) (N = 21; LSD method); * above bars indicate a significant difference between the females and males at P <0.05 (F-test, N = 6); ** indicate extremely significant difference at P <0.01 (F-test, N = 6).

Generally, males of most butterflies are more active than females during flower visits. However, both males and females of *P*. *xuthus* were very active during foraging. In the absence of any chemical cue, both females and males mainly visited blue (440–475 nm, 47%), purple (380–420 nm, 27%), and red flowers (615–630 nm, 15%) (Fig 3A), response to these three colors is significantly higher than other colors (F = 468.04; N = 6; P = 0.000; α = 0.01). In these three colors, blue is higher than purple and red (F = 120.066; N = 9; P = 0.000; α = 0.01). Together, 84.97% of female flower visits and 94.11% of male flower visits were to blue, purple, and red flowers. The preference of females and males toward flowers with these three colors was obviously different (F = 15.077; N = 6; P = 0.018; α = 0.05). The percentage of females visiting the blue flower was 28 (54.90%), and the percentage for the males was 20 (39.22%). The percentage of females visiting the red flower was 5 (9.80%), and that for the males was 10 (19.61%); the percentage of females visiting the purple flower was 10 (19.61%) and that for the males was 18 (35.29%) (Fig 3A).

Chemical cues had a significant impact on butterfly behavior. After spraying 10% honey water on otherwise odorless, artificial flowers, the number of visiting flowers dramatically increased, the total number of flower visits increased 3.34 times (F = 275.208; N = 6; P = 0.000; α = 0.01). More specifically, visits to flowers with honey water by females and males increased 3.41 and 3.25 times, respectively, in comparison to the same flowers without honey water, there were no significant differences for female and male visiting flowers (F = 1.550; N = 6; P = 0.281; α = 0.05) (Fig 3C). Further, the total number of butterfly visits significantly increased 37.89 fold (F = 564.948; N = 6; P = 0.000; α = 0.01) to flowers applied 10% honey water plus colors in compared to honey water alone controls (Fig 3C), Males were more sensitive to flower and honey combinations than females (F = 10.878; N = 6; P = 0.03; α = 0.05), with 83.50 times increase in male visits and 24.86 times increase in female visits. However, compared to visit colorful flowers without honey water, the total number of visiting flowers increased only 3.34 fold in the experiment of color plus odor (Fig 3C), indicating that colors played a more important role than odor, although *P*. *xuthus* relied on both visual and chemical cues during foraging.

### Response to color and odor during courtship

**Fig 4:**
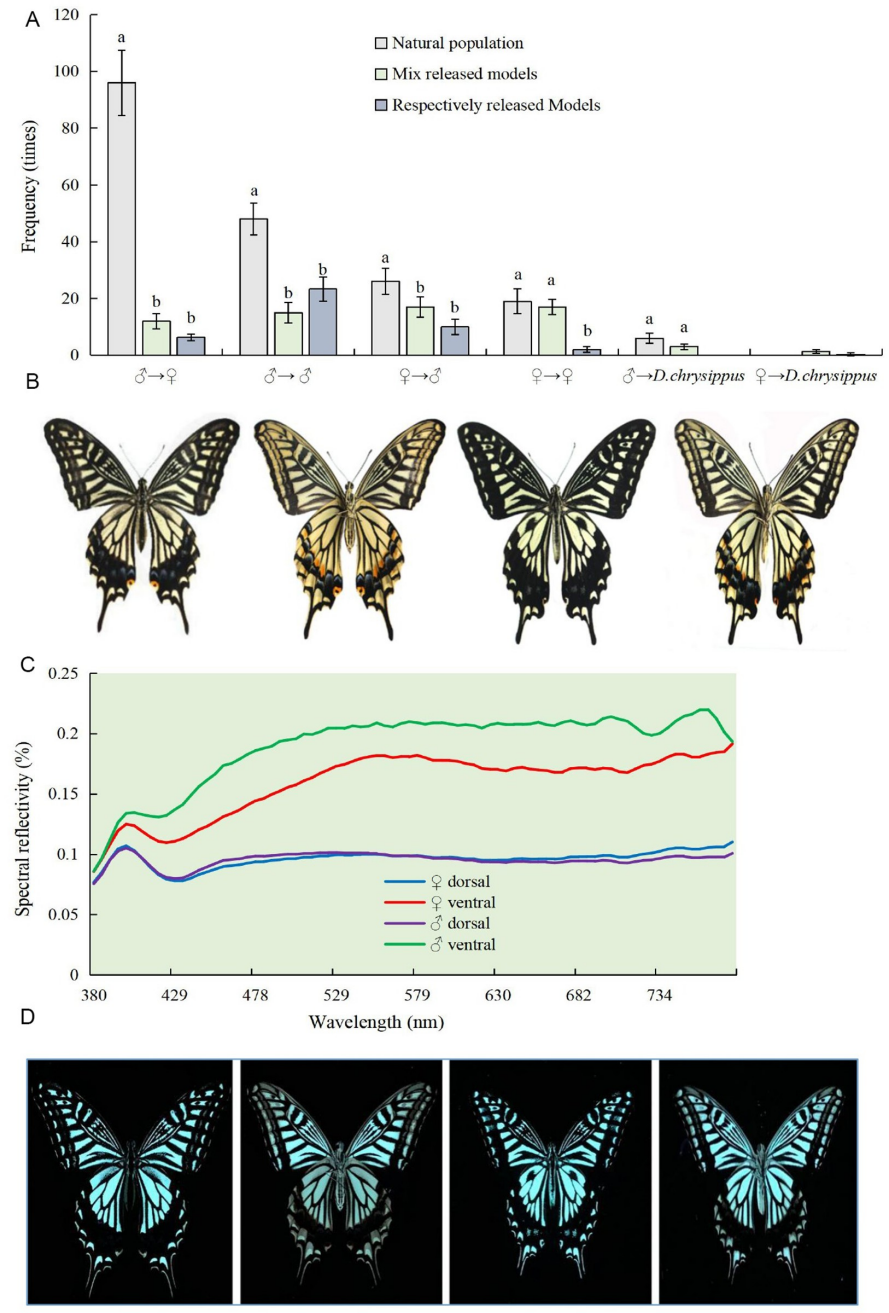
Chase behavior of *P*. *xuthus* during courtship and wing reflection spectrum. A. Chase behavior of *P*. *xuthus* during courtship using both natural butterflies and butterfly models. B. Representative butterfly specimen under natural light. C. Reflective spectrum of the butterfly specimen. D. Images of butterfly specimen under UV light. Notes: Histograms with alphabet above bars indicates significant difference under the same chasing behavior at P <0.05 (N = 9; LSD method).

During courtship, four types of chasing behavior of natural *P*. *xuthus* populations were observed. Namely, males chasing females, males chasing males, females chasing males, and females chasing females (Fig 4A). Nearly half (49%) of chasings were males chasing females, but only 13% of chasings were females chasing males. Interestingly, 25% of chasings were between males and 10% between females. On the other hand, *P*. *xuthus* hardly chased the other butterfly, *D*. *chrysippus*. This indicated that *P*. *xuthus* could identify conspecific butterflies and reject the heterogeneous butterflies in nature (Fig 4A).

When butterfly models were used, their chasing models had obvious differences compared with the natural populations (F = 84.066; N = 12; P = 0.000; α = 0.01). Mix released models, the frequency of males chasing females was 12 (18%), whereas females chasing males was 17 (26%). On the other hand, the frequency of males chasing males was 15 (23%), and females chasing females was 17 (26%) (Fig 4A). Respectively released models, the frequency of males chasing female models was 6 (14%), whereas the frequency of females chasing male models was 10 (24%). On the other hand, the frequency of males chasing males was 23 (56%), and females chasing females was 2 (5%) (Fig 4A). Unlike in the natural population, model chasing cannot precisely distinguish sex partners in the mixed model and respective model. However, *P*. *xuthus* hardly chased *D*. *chrysippus* models, indicating that *P*. *xuthus* butterflies can identify heterogeneous conspecies butterflies but could not distinguish females and males of their own if only patterns of colors are available.

### Analysis of wing color spectrum

It is very difficult to distinguish females from males of *P*. *xuthus* because their color and stripes are very similar (Fig 4B). The color spectrum of wings from both males and females is in the range of visual and ultraviolet light, with only a few differences observed on the ventral surface of the wings (Fig 4C and 4D). These results may explain why these butterflies could not recognize their partners by vision alone during courtship.

### Body VOCs and their roles in courtship

**Fig 5:**
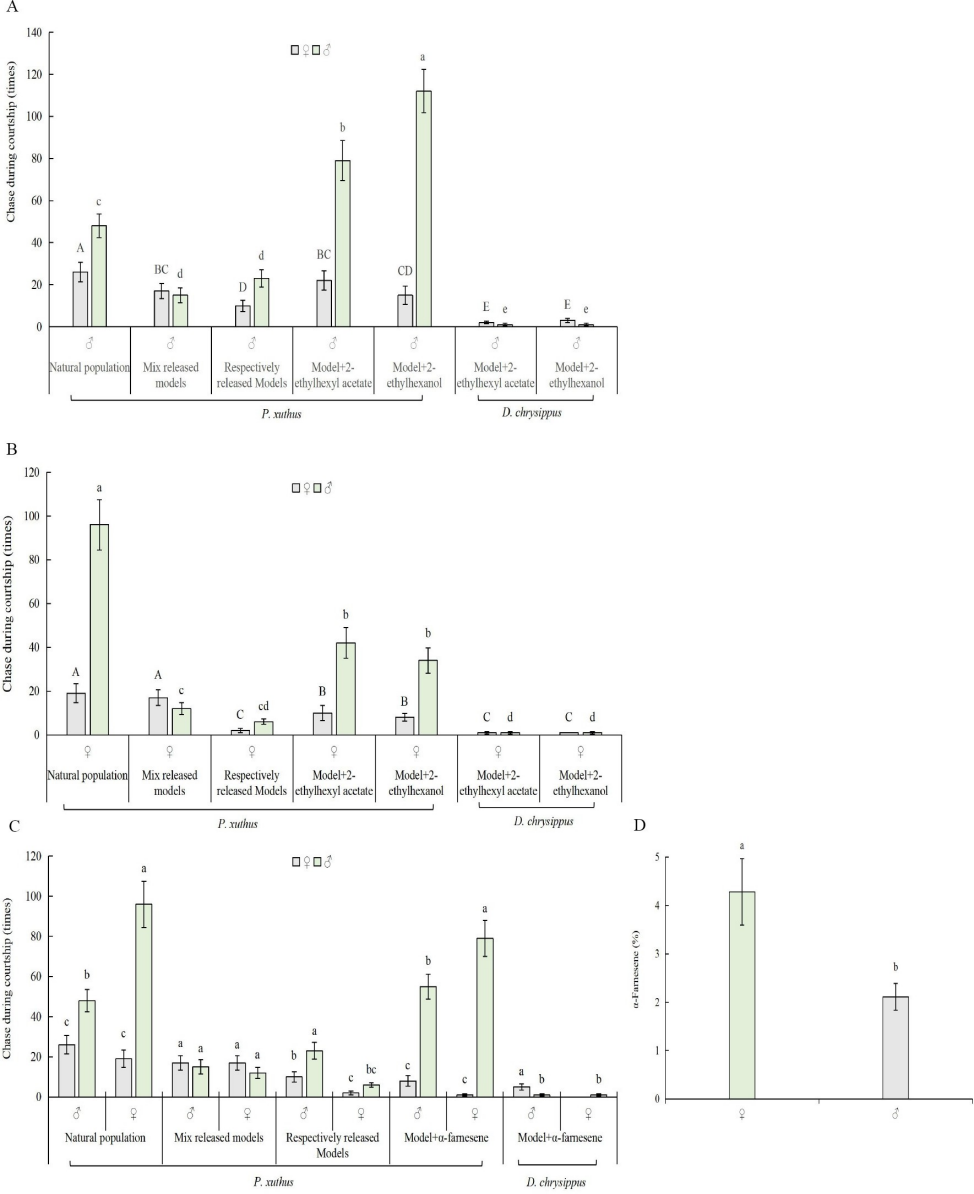
Chasing behavior of the butterfly *P*. *xuthus* to VOCs during courtship. A. Chasing number between males and females to butterfly males models applied with 2-ethylhexyl acetate and 2-ethyl-1-hexanol. B. Chasing number between males and females to butterfly females models applied with 2-ethylhexyl acetate and 2-ethyl-1-hexanol. C. Impact of α-farnesene on the chasing behavior of the butterflies: *P*. *xuthus* and *D*. *chrysippus*. D. Abundance of α-farnesene in females and males of *P*. *xuthus*. Notes: Capital letters indicate significant differences between the female butterfly chasing the model (P<0.05) (N = 21, LSD method) in Fig 5A, 5B, lowercase letters indicate significant differences between the male butterfly chasing model (P <0.05) (N = 21, LSD method) in Fig 5A, 5B. Lowercase letters indicate significant differences between the four types of chasing behavior (P <0.05) (N = 12, LSD method) in Fig 5C. Different lowercase letters above bars indicate significant differences in the abundance of α-farnesene between males and females (P <0.05) (F-test, N = 6) in Fig 5D.

During courtship, chemical cues play important roles in identifying sex partners in insects, especially in butterflies, because females and males of butterflies are very similar in patterns of both color and shape. We measured 29 VOCs obtained from *P*. *Xuthus* individuals to examine potential chemical cues. The 29 VOCs belonged to 11 different categories, and no sex-specific VOCs were observed in females or males (Table 1). The top three categories of the identified VOCs included alcohols (♀ 38.39%; ♂ 37.52%) and esters (♀ 34.19%; ♂ 32.15%). We selected three abundant chemicals, 2-ethylhexanol (alcohol), 2-ethylhexyl acetate (ester), and the aromatic α-farnesene, to test their ability to attract butterflies during courtship. The total number of males chasing models increased significantly after spraying the three chemicals (F = 10.367; N = 15; P = 0.001; α = 0.01). However, except for α-farnesene, no significant increase was observed in the number of males chasing female models after spraying 2-ethylhexanol, 2-ethylhexyl acetate (Fig 5A–5C). On the other hand, *P*. *Xuthus* butterflies hardly chased *D*. *chrysippus* models. However, the three VOCs were applied on the heterogenous models (Fig 5A–5C), suggesting that patterns of colors were the main cues in identifying heterologous conspecies butterflies.

**Table 1:** Classification of VOCs in the adults of *P*. *xuthus*.

Kinds of VOCs	Contents (%)	
	♀	♂
Alkanes	5.1	4.19
Aromatic hydrocarbons	1.54	1.38
Alcohols	38.39	37.52
Aldehydes	1.6	0.25
Ketones	1.3	1.24
Lipids	34.48	32.31
Terpenoids	4.65	10.54
Acids	3.65	3.45
Phenols	0.15	0.22
Ethers	7.39	7.10
Heterocyclics	0.22	0.10

Butterflies chased butterfly models in a way similar to that observed in the natural population during courtship after the models were sprayed with α-farnesene (Fig 5C). The content of α-farnesene in females was higher than that in males 3–5 days after emergence (Fig 5D); the high abundance α-farnesene might be responsible for distinguishing males from females in natural butterfly populations. On the other hand, the number of males chasing *P*. *xuthus* models increased when we sprayed VOCs on the *P*. *xuthus* model (Fig 5A–5C), it could be because a male is more initiative than a male-female during courtship and color plus VOCs enhanced recognition ability to homogeneity.

## Discussion

Lepidopterans generally use multi-modal sensory cues during nectar foraging, but the integration of such cues may be complex and hierarchical [27, 28]. Tang [2] pointed out four types of signal perceptions during butterfly foraging and courtship, including perception based predominantly on vision, perception based predominantly on olfaction, perception based equally on vision and olfaction, and perception on olfaction alone. To examine the signal perception mechanism of the butterfly *P*. *Xuthus*, we tested the responses of *P*. *xuthus* to visual cues, chemical cues, and the combination of visual and chemical cues. We found that *P*. *xuthus* used primarily visual cues during flower visits when visual cues and chemical cues were separated. However, synergism was observed when visual and chemical cues were present together during flower visits. Using visual cues as the predominant signals during foraging by butterflies may be because colorful flowers are easy to identify in nature.

On the other hand, chemical cues from VOCs in nature are abundant, complex, and unstable, which may need to invest more resources in identifying and distinguishing the information from various VOCs. The ancestors of butterflies could primarily look for food resources by odor cues before phanerogam; the emergence of colorful flowers could drove butterfly to develop visual system because color provides more obvious cue of food information than odor during foraging, in long evolution, butterfly interacted and coevolved with flowers resulted in colorful butterfly. During this process, butterflies may have gradually evolved and developed visual perception as the main signal recognition due to less cost in finding foods and distinguishing sex partner.

Butterflies use a variety of visual and chemical cues to identify the opposite sex during courtship [29–31]. Most butterflies first use visual signals to distinguish females and males and then use chemical signals to differentiate them further [31–33]. Our results indicated that chemical cues are precise information to recognize the opposite sex in the near distance during courtship. *P*. *xuthus* could accurately distinguish individual butterflies from other species solely based on visual cues. However, visual cues were not enough to distinguish males from females of its own species, likely due to very similar colors and patterns of wings on both *P*. *xuthus* males and females. The frequency of males chasing females was only 19% in the absence of chemicals. The frequency of males chasing females increased to 40% when VOCs was applied, suggesting that chemical cues played an important role in identification of opposite sex during courtship.

Possible chemical cues include sex hormones and other body VOCs [17–20, 33–34]. Analyses of VOCs on the butterfly *P*. *xuthus* identified several abundant VOCs, which could play roles in the identification of mates during courtship. Several VOCs with quantitative differences between males and females were found. The quantitative differences in VOCs might serve as cues for chemical perception during mating. Indeed, the application of two abundant VOCs, 2-ethylhexanol and 2-ethylhexyl acetate, resulted in increased total courtship chases among *P*. *Xuthus* individuals. However, the application of the aromatic volatile α-farnesene resulted in an increased frequency of males chasing females like natural population courtship. In this experiment, we found that the content of VOCs was not closely related to recognizing sex partners; however, the time of chasing model increased after spraying these VOCs on models, indicating that the high content of VOCs is beneficial in recognizing homogeneity. Interestingly, the frequency of male chasing female models increased after spraying α-farnesene, implying that aromatic volatile could be an important cue for *P*. *Xuthu* to distinguish both sexes during courtship, it could be that α-farnesene was easy volatilizing and higher content of female than male. *P*. *xuthus* hardly chased *D*. *chrysippus* butterflies. The spray of these three chemicals from *P*. *xuthus* on heterogenous butterfly *D*. *chrysippus* model indicated that *P*. *xuthus* could distinguish it from other species depending on colors even though its volatiles were sprayed on other species.

## Supporting information

S1 Data (ZIP)
